# Loneliness, Spirituality, and Health-Related Quality of Life in Hispanic English-Speaking Cancer Caregivers: A Qualitative Approach

**DOI:** 10.1007/s10943-023-01880-x

**Published:** 2023-09-04

**Authors:** Jennifer J. King, Terry A. Badger, Chris Segrin, Cynthia A. Thomson

**Affiliations:** 1 University of Arizona Cancer Center, University of Arizona, 1295 N. Martin Ave., Drachman Hall, A260, PO Box 245209, Tucson, AZ 85719 USA; 2 College of Nursing, University of Arizona, Tucson, AZ USA; 3 College of Social and Behavioral Sciences, University of Arizona, Tucson, AZ USA; 4 Department of Health Promotion Sciences, The University of Arizona Cancer Center, University of Arizona, Tucson, AZ USA

**Keywords:** Caregiver, Hispanic, Cancer, Loneliness, Spirituality

## Abstract

Hispanic caregivers experiencing higher caregiving burden than their non-Hispanic cohorts, due in part to contextual factors, such as barriers to accessing health care, challenging employment environments, low education and income, immigration issues, and minority stress. Spirituality may serve as a coping strategy for Hispanic caregivers that influences health-related quality of life (HRQoL), possibly by modifying loneliness associated with caregiving. We explored these concepts using semi-structured interviews (*N* = 10 Hispanic caregivers). Participants shared perceptions of loneliness, spirituality, and how these factors related to HRQoL. Five themes emerged: caregiver experience, coping strategies, loneliness, religion and spirituality to gain strength. Findings suggested that spirituality and religion improved HRQoL partially by reducing loneliness. Future programs to improve HRQoL in Hispanic English-speaking cancer caregivers should address spirituality.

## Introduction

Culturally competent approaches (sensitivity to cultural norms, spiritual activities, or self-care practices) are needed to improve quality of life for informal caregivers of the estimated 149,100 new Hispanic/Latinx/o/a cancer patients annually in the USA (American Cancer Society, 2021a, b). In this paper, Hispanic/Latinx/o/a are used to refer to persons of Hispanic origin and are used interchangeably without preference or prejudice (American Cancer Society, 2021a). Informal caregivers are defined as family members or friends who provide unpaid supportive, informational, and emotional care (National Alliance for Caregiving, 2019) and perform palliative care responsibilities (Miller et al., 2014; Vitaliano et al., 2003). The psychosocial needs and related quality of life of Hispanic caregivers of cancer patients are not well understood. With a growing survivorship population overall and poorer outcomes for several cancers among Hispanic patients, the need for caregiving is expected to rise. Caregivers encounter challenges when meeting caregiving demands, and these challenges are associated with changes to both their personal and other aspects of their life (Vitaliano et al., 2003).

### Hispanic Caregiving and Cultural Values

Caregiving is a culturally embedded value among Hispanics due to the concept of *familism*, that places a higher emphasis on the family unit over the individual in terms of respect, support, and obligation (Valdivieso-Mora et al., 2016). Compared to non-Hispanic white (NHW) families, informal care among minority families is often more prevalent with Hispanics (Rote & Moon, 2018), who regularly experience higher burden from caregiving and spend more time caregiving (National Alliance for Caregiving, 2019). Hispanic caregivers generally provide 45% of all caregiving tasks, with an estimated 30 h per week in high burden caregiving situations (National Alliance for Caregiving, 2019). Contextual factors, such as barriers to accessing health care, challenging employment environments, low education and income, immigration issues, and minority stress are often encountered by Hispanic caregivers (Badger et al., 2019). These factors are encountered within the context of several cultural influences.

Overtly, due to cultural gender role expectations, women are traditionally more likely to provide care for family members with cancer (Jolicoeur & Madden, 2002). Similar to our findings, Heath et al. (2018) study had disproportionately represented women as familia caregivers (Heath et al., 2018). The intersectionality of being both female and Hispanic, coupled with such cultural gender role expectations potentially puts an extensive burden on female caregivers. These women are more likely to have less education, lower income, and work in jobs with fewer benefits (Badger et al., 2019; Kayser et al., 2021). Hispanic cancer caregivers report high distress and reduced health-related quality of life (HRQoL) (Badger et al., 2013; Segrin et al., 2019). Greater distress is sometimes attributable to language barriers (e.g., not being fluent in the dominant language of the culture) or social determinants of health (i.e., lack of access to services, fewer recreational and leisure opportunities, lack of available transportation, lower incomes and education) (Treiman et al., 2021). Hispanic caregivers often report more unmet needs concerning psychological and information support (Ashing-Giwa, 2005), in part due to language barriers, stigma about seeking help outside the family or church, mistrust of institutions or lack of access to supportive care (Chebli et al., 2020).

### Loneliness

Loneliness, commonly defined as a perceived discrepancy between one’s desired and achieved levels of social contact (Peplau et al., 1979), is a psychosocial symptom associated with lower HRQoL (Hawkley & Cacioppo, 2003, 2010; Segrin et al., 2021). Loneliness is a problem for not only the survivors but for caregivers (Soylu et al., 2016). Caregiving frequently generates demands that limit discretionary social contacts thus increasing loneliness (Beeson, 2003). Hawkley and Cacioppo (2010) theorize that loneliness accelerates age-related declines in health through mechanisms such as intensified stress responses and dysfunctional health behaviors (Hawkley & Cacioppo, 2003, 2010; Segrin et al., 2021). Addressing loneliness using culturally appropriate methods may potentially improve HRQoL among the Hispanic caregiving population. The impact of loneliness on quality of life may be more complex and driven by related contextual factors unique to Hispanic caregivers.

### Spirituality

Spirituality has been identified as an important dimension of quality of life in cancer survivorship (Lalani et al., 2018). For Hispanics, health issues are often met with a spiritual approach of prayer and reliance on faith, as religiosity and spirituality are important traditional values, (Lujan & Campbell, 2006; Rehm, 1999). Religiosity comprises organizational approaches (e.g., attendance at a church or organized entity), behaviors and practices related to a specific doctrine. Spirituality consists of a relationship between God or a higher power, one’s self, community, and one’s environment (Campesino & Schwartz, 2006; Moberg, 2020). Spirituality among Hispanics includes personal prayer and reliance on religious faith for health issues (Lujan & Campbell, 2006; Rehm, 1999). Hispanics who regularly go to church and those who attend only sporadically are equally likely to identify prayer as important for the health of family members (Ransford et al., 2010). Religiosity is an important coping mechanism for Hispanic caregivers that is negatively associated with their level of anxiety (Gonyea & O’Donnell, 2021).With spirituality and religiosity being recognized as a pathway to health and well-being, these factors may be foundational sources of support and considered to be a positive coping practice among Hispanic caregivers (Herrera et al., 2009).

Within the Hispanic culture, a relevant value is *espíritu*—trust in spirituality. Explicitly, *espíritu* is an important Hispanic core cultural value in which one may find solace. Interestingly, Hispanics with higher spirituality report lower levels of loneliness (Gallegos & Segrin, 2018). Similarly, intrinsic and organizational religiosity has been associated with lower perceived burden, while non-organizational religiosity was associated with poorer mental health (Herrera et al., 2009). A concept often associated with Hispanic culture is religious fatalism. In contrast to spirituality and religiosity, religious fatalism is linked to poorer health outcomes and reduced healthy behaviors among Hispanic and African American communities, particularly through pathways of diminished agency and passivity in health decision-making (Franklin et al., 2008; Leyva et al., 2014).

### Future Approaches

In summary, culturally valued constructs among Hispanics such as spirituality and religiosity may serve as strategies to help Hispanic caregivers cope with the psychosocial risk and related diminished HRQoL outcomes associated with cancer caregiving (Nance et al., 2018). These strategies hold promise to influence loneliness, a driver of poor HRQoL. Few studies have assessed caregivers’ views concerning loneliness, spirituality and HRQoL, particularly in Hispanics. Therefore, understanding these experiences will inform program development to improve HRQoL among Hispanic cancer caregivers. With limited psychosocial interventions that successfully target and support Hispanic cancer caregivers (Borrayo et al., 2020), understanding their experiences will aid in creating supportive care interventions, address challenges, and provide needed support.

## Methods

Ten Hispanic caregivers of cancer patients described their perceptions of loneliness and spirituality, and how loneliness and spirituality related to their HRQoL. Participants were recruited from a large, randomized control trial investigating symptom management interventions, [National Institute of Health (NIH), R01CA224282]. For the purposes of this qualitative descriptive study, study participants from the parent study who elected to be contacted for future studies were contacted by the trained study coordinator. All previously enrolled caregivers who were (1) a caregiver of a cancer survivor with a diagnosis of cancer of any type; (2) ≥ 18 years at the time of participation; and (3) self-identifying as Hispanic, and who represented a range of scores (low, medium, high) on the PROMIS social isolation-short form 4a baseline were invited to participate. Following IRB approval [2007861602] and informed consent, caregivers were asked to participate in a 30–90 min telephone semi-structured interview. All interviews completed were in English as this was an inclusion criterion for the institutional review board which approved this study, although most participants were bilingual Spanish and English speakers.

### Semi-structured Interviews and Qualitative Data Processing

The telephone interviews were conducted in English and consisted of open-ended questions and prompts identifying the participants’ perceptions of loneliness, spirituality, and health-related quality of life related to cancer caregiving (Appendix . Interview Guide). The interview guide was developed by the research group and was informed by prior quantitative, self-report questionnaire responses from Hispanic cancer caregivers in the parent study related to HRQoL, as well as a review of literature (Badger et al., 2019). The interview was guided by the pre-planned questions and or prompts. However, the researcher had the flexibility for follow-up questions and or additional prompting as necessary. Specifically, when the interviewer (JM) did not understand what the participant stated during the interview, prompts were used such as “can you explain that more, I don’t want to assume.” The interviews were audio recorded, transcribed, stripped of any identifying information, and downloaded into the qualitative data software. MAXQDA 2020 was utilized to organize the data (VERBI Software, 2019). Interviews and analyses were simultaneously conducted with interviews checked for accuracy by JM and TB. The authors comprise two researchers from public health, one from nursing, and one from communication. All have experience with the collection and analysis of qualitative data as well as with family caregiving for people with cancer, and two have extensive backgrounds with Hispanic cancer survivors and caregivers.

Braun and Clarke’s (2006) process of thematic analysis was used to analyze the data (Braun, 2006). This process included (a) interviewing, transcribing, and reading as well as re-reading the data to gain familiarity with them; (b) identifying portions of the data answering the research questions that were coded (highlighting sections of text, typically phrases or sentences, and creating labels or “codes” to describe their content); (c) collating all data into groups, identified by codes, into initial minor categories, which were broader than codes; (d) refining minor categories to ensure they were representative of the data set; and (e) selecting exemplars for each theme. Finally, through an iterative process of comparing categories with the primary (JM) and secondary coder (TB), the data were queried for new categories or themes related to the experiences from the Hispanic cancer caregivers. When no new themes emerged, it was concluded that saturation was reached.

Four criteria of credibility, transferability, dependability, and confirmability were applied to evaluate the trustworthiness of the findings (Lincoln & Guba, 1985). Credibility, or truth in the findings, was maintained by using direct transcribed quotes from the participants reviewed by the secondary coder (TB) for interviews one, three, eight, and nine. Transferability, or that the findings have applicability in other contexts for similar samples, was maintained by the data collection records and decision trails being kept by both reviewers JM and TB. Dependability, or that findings are consistent and may be repeated, was maintained by ensuring that all processes were recorded, reviewed by the secondary coder (TB), and both (JM and TB) kept an audit trail. Confirmability, or a degree of neutrality, was maintained by ascertaining participants’ perceptions to any statements that were unclear to the interviewer with follow-up probes that were devoid of any leading qualities.

## Results

Descriptive statistics are presented in Table 1. The mean age of caregivers in the study was 58.8 years of age; 60% were ≥ 60 years of age (Table 1). Half the participants were married. Seven of the ten caregivers had a college education or some technical education. In total, participants were employed full time (5), retired (3), employed part time (1), or not employed (1). The majority were female and were spouses/significant others (*n* = 2) or relatives (*n* = 4) to the survivor.

**Table 1:** Descriptive statistics

Variable	(*N* = 10) mean (SD) or *N* (%)
Age	58.8 (13.7)
Ethnicity (Hispanic)	10 (100%)
*Marital status*	
Married	5 (50%)
Single/not married	5 (50%)
Sex of participant (female)	6 (60%)
*Relationship to survivor*	
Spouse or significant other	6 (60%)
Child (daughter)	3 (30%)
Mother	1 (10%)
*Education*	
Less than high school	1 (10%)
High school	1 (10%)
Vocational/technical school or some college	3 (30%)
College	4 (40%)
Postgraduate	1 (10%)
*Employment*	
Full-time	5 (50%)
Part-time	1 (10%)
Unemployed, seeking	1 (10%)
Retired	3 (30%)

**Fig. 1:**
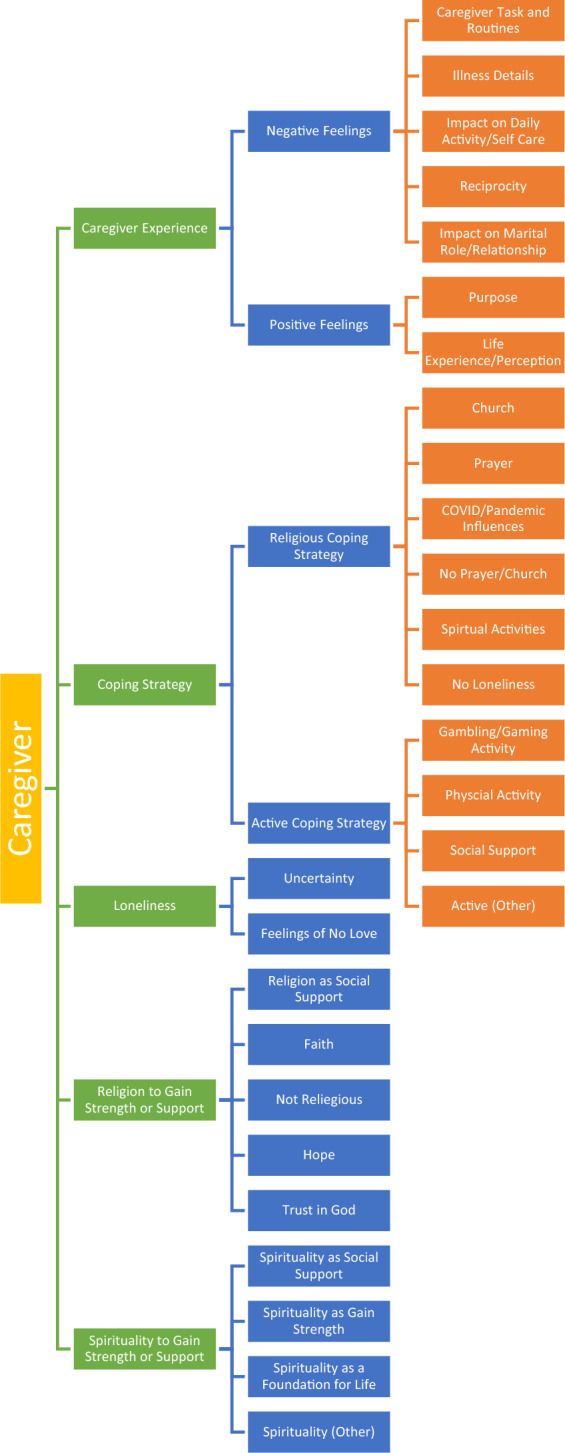
Themes, major and minor categories

The five themes (caregiver experience; coping strategy; loneliness; religion to gain strength or support; spirituality to gain strength or support), four major categories (negative caregiving feelings; positive caregiving feelings; religious coping strategies; active coping strategies) and 17 minor categories (presented in Table 2 and Fig. 1) provide insight into Hispanic cancer caregivers’ perceptions of their caregiving.

**Table 2:** Themes, major and minor categories with exemplars

Themes	Major categories	Minor categories	Exemplars
Caregiver experience	Negative feelings	Caregiver task and routines	“I communicate with her consistently. I go to the appointments, probably 90% of the time that she goes with her. I know I, I have, I have a record of what her last visit was like, what came up, where her tumor markings are at, what has changed from the last six months, when her next PET scan.” (#7)
		Illness details	“I would take her to the hospital and sit there with her while she had her treatments done. We realized pretty soon that it was terminal and she volunteered for tests. She became extremely dependent upon me. She didn’t want me to leave the room. She didn’t want to go out anywhere. She was afraid to fall asleep because she was afraid to wake up and I wouldn’t be there. It really became intense.” (#2)
		Impact on daily activity/self care	“It has a great impact on one’s life, in my case. I mean, if I want to go now on vacation, I won’t be able to go with her on vacation because she has that disease of the diarrhea because of the scleroderma, you know, and that we have to watch out when we go out and where we are going out.” (#9)
		Reciprocity	“And one more thing. Yeah. I think, you know, when you become a caregiver for all those years, whether it’s the spouse or a brother that you have to take care of or whoever it is in the family that you have to constantly take care of. It gets to the point that sometimes they take it for granted.” (#9)
		Impact on marital role/relationship	“It’s, I’ve learned, I’ve had to learn how to put boundaries. So therefore, before I felt a sense of obligation because I was his girlfriend, I was his ex-girlfriend, I was his only person that knew the real situation. I always felt obligated.” (#4)
	Positive feelings	Purpose	“It made me happy to take care of her. It didn’t, the stuff I couldn’t do didn’t bother me at all. Yeah, she was my main concern. She was my life. Anything I could do ease her pain.” (#2)
		Life experience/perception	“And just getting to know all the whole, other side of my daughter you know, that closeness that you have with a daughter it’s always there, but when there’s an illness involved, oh my God, that was so much bigger.” (#3)
Coping strategy	Religious coping strategy	Church	“For me going to church is important. Even though, you know, there’s time, to me, I been limited just depending on what my daughter and how she’s feeling.” (#3)
		Prayer	“You know, as far as activities. No, I have not, but the prayers are, my prayers are daily. Yes. My prayers are morning and in between the days, if I feel I’m having to need one and at night times, always, always. Yeah. But activities [religious], really no.” (#3)
		COVID/pandemic influences	“And now with this, COVID, there’s been a lot, a lot of, it’s been a really hard, it’s been really, really, really hard, and there’s a lot of us that are calling each other and, how are you doing? You know, because that’s, we see each other once a year [Sundance]. And it’s really difficult. It’s really, really difficult.” (#4)
		No prayer/Church	“So, I’m not very big on organized prayers saying, you know, the same thing over and over again to me that doesn’t make any sense.” (#2)
		Spiritual activity	“Loneliness for me is not as terrible as what I know other people to suffer, but because like, meditation, you know, is an inward activity. So, it helps you be with yourself and religious activity, and prayer and participating in other groups like in prayer groups, which you have, it helps. It is for me; it is both of those activities are quite important.” (#5)
		No loneliness	“No, I don’t, I’ve never had those situations. I don’t allow my mind to get into that hole there. No, because I’m active and I try to be active and busy. So I don’t get into that hole. No, I refuse to do that.” (#9)
	Active coping strategy	Gambling/gaming activity	“If I ever feel really lonely, I tell them, take me to the casino and that wakes me up.” (#6)
		Physical activity	“For example, I may go do a hike. Then, you know, I’ll do it for like three months” (#7)
		Social support	“Communicating with my siblings about what was going on. I think is well, keep maintaining that balance in my life.” (#7)
		Active (other)	“I used to play the guitar and sing in Spanish and that would help me so much, you know, when I would get my guitar…I haven’t played, played my guitar lately. That would take the loneliness away from me.” (#6)
Loneliness		Uncertainty	“And during the treatments, I was always with her. So, we were kind of sharing those feelings of uncertainty and worry. We shared and helped each other get through it. So, we fortunately were able to do that. So, we got through it.” (#5)
		Feelings of no love	“I feel like I’m not good enough, very low self-esteem. And now like nobody’s here for me. That’s what I feel. Like you’re not loved, like you are, you don’t have it to hold on to. I feel ugly sometimes when I feel that way [loneliness], because those are the talks that come into my mind like, Oh, you are on underserving.” (#8)
Religion to gain strength or support		Religion as social support	“It has opened up for other ways served. So, because, because of my background I am able to help with other things around the church that are not the typical ministries. There’s the technical aspect of streaming all these activities and I’m able to help in there as well. So that’s open somewhere else where we can serve.” (#5)
		Faith	“Well, because you hope that the faith that you have will continue to be there for you and just one day at a time.” (#9)
		Not religious	“But even then, I joke that I was too old for it to really have a grip on me because, you know, I got into it too late. So, I don’t mean, although I love the general aspects, I might believe I don’t, you know, there’s a lot of details that I don’t.” (#10)
		Hope	“That’s a hope but we are real people and I understand that is the diagnosis and I’m just, you know, what is changed is just the hope that she gets better and she doesn’t, you know, she will be in a better place when she passes.” (#8)
		Trust in God	“I try to tell her. But again, I know, I know that one day God’s going to heal her for me. He [God] is. And I just can’t wait for that day, what rejoicing that will be.” (#3)
Spirituality to gain strength or support		Spirituality as social support	“But in general, being spiritual and appreciating things and feeling like that’s definitely a component and the things we’ve gone through, and helping us out, I think that’s, it’s been stronger. Like it’s gotten stronger, but not necessarily the participation in that church, you know?” (#10)
		Spirituality as gain strength	“To classify us in general, we’re not the most religious, we’re not the most religious. And we haven’t been like prior, but I’d say really, if we were more like agnostic, especially me like my position, but I’d say, and that’s like, just since forever. So, but now really, I mean, I’d say if any time we’ve been stronger or more kind of spiritual, it’s has been stronger now than it probably ever has been.” (#10)
		Spirituality as a foundation for life	“So, I’m not a religious person at all. I don’t, however we are, we’re a very strong spiritual person. So, there’s kind of a difference in that. I have a hard time conforming to manmade religion versus to just the spirituality.” (#4)
		Spirituality (other)	“So, I’ve done yoga in the past, but it’s been for short periods of time. I have been more, I have been putting more time into the meditation practice, then I have to go do yoga.” (#7)

### Theme 1—Caregiver Experience

The first theme concerned the caregiver experience, which was defined as both the negative and positive feelings related to caregiving; changes in roles and responsibilities; details about treatment and illness of the survivor after diagnosis; influences of the survivor’s illness on the caregiver’s daily life and self-care; reciprocity related to social support from the care recipient and family members; influences of the survivor’s illness on marital roles and relationship between the survivor and caregiver; and influences of the survivor’s illness on caregivers’ life experience and perception. Two major categories related to negative and positive feelings emerged. The category of negative feelings included: (a) caregiver task and routines; (b) illness details; (c) impact on daily activity/self care; (d) reciprocity; and (e) impact on marital role/relationship. Positive feelings included: (a) purpose and (b) life experience/perception.

A number of participants discussed the burdens of caregiving. One of the most important issues for caregivers who are caring for someone with a chronic progressive disease is the burden of care. The burden of care causes the caregivers to put off meeting their own needs, which leads to unpleasant experiences such as disrupted interpersonal relationships and poor physical health, all of which have an impact on the caregiver’s well-being and can lead to increased stress. One participant explicitly mentioned negative feelings, specifically stress: “Well the caregiving, I think to me it’s a little, not a little, but I think it needs more understanding of what the caregivers go through, but it also depends on what the qualities of a person might be. You know, she doesn’t only have cancer or right now she’s cancer free, but she also has other chronic diseases. So that makes a caregiver really, really extremely stressed. They don’t know how to cope with the stress.” (#9).

Increased stress and anxiety are the most typical signs of emotional issues reported by caregivers. Another participant expressed anxiety related to the caregiving experience as: “So for me, it’s, I mean, I feel, I felt good and happy that I could help her and do things like that. But at the same time, it’s created more anxiety for me. And I know like I have issues with OCD, so I know it’s exacerbated that. So, I don’t think I’ve really taken enough time to kind of work on maybe my self-care in that respect, like with anxiety and I have to some degree, but I know I can do more, but like being anxious and kind of worrying about things. So, I’d say it’s made me more anxious, you know?” (#10).

Caregivers go through a range of emotions as a result of their roles. Some those feelings are negative. Feelings of frustrations, diminished stamina, and a decrease in one’s self-esteem comprehensively led to negative feelings. Negative emotions related to the survivor’s current health status were similarly expressed, “I would take her to the hospital and sit there with her while she had her treatments done. We realized pretty soon that it was terminal and she volunteered for tests. She became extremely dependent upon me. She didn’t want me to leave the room. She didn’t want to go out anywhere. She was afraid to fall asleep because she was afraid to wake up and I wouldn’t be there. It really became intense.” (#2).

Becoming a caregiver has numerous unexpected changes to one’s life. Change to the caregiver’s life were expressed by another participant as, “It has a great impact on one’s life, in my case. I mean, if I want to go now on vacation, I won’t be able to go with her on vacation because she has that disease of the scleroderma, you know, and that we have to watch out when we go out and where we are going out. You know, they don’t realize that a caregiver is going through hell as well, and that should be stressed to the patients as well.” (#9).

While caregiving is a dynamic experience over time with ever increasing complexity and scope of responsibilities, positive experiences are often encountered as well. Caregiving as a positive emotion was expressed as well, “It made me happy to take care of her. It didn’t, the stuff I couldn’t do didn’t bother me at all. Yeah, she was my main concern. She was my life. Anything I could do ease her pain.” (#2). Another caregiver expressed her caregiving as a positive life experience “And just getting to know all the whole, other side of my daughter you know, that closeness that you have with a daughter it’s always there, but when there’s an illness involved, oh my God, which was so much bigger.” (#3) Table 2 presents additional exemplars of the themes.

### Theme 2—Coping Strategy

All caregivers discussed the emerged theme of coping strategies related to loneliness and the caregiving role. This theme was categorized into two major categories being (1) active coping strategies and (2) religious activity coping strategies. Active strategies encompassed physical activity (hiking, running), leisure (gambling, playing instruments), and finally social support (seeking out time with friends and family). Religious coping strategies included any religious activities such as going to church, Bible studies, praying, and meditation, all of which were referred to by the participants. One caregiver expressed that “For me going to church is important. Even though, you know, there’s time, to me, I been limited just depending on what my daughter and how she’s feeling.” (#3).

Alternatively, when asked about specific strategies, prayers were revealed as a coping strategy among most of the participants “You know, as far as activities. No, I have not, but the prayers are, my prayers are daily. Yes. My prayers are morning and in between the days, if I feel I’m having to need one and at night times, always, always. Yeah. But activities [religious], really no.” (#3) When discussing spiritual coping strategies related to caregiving loneliness, one caregiver detailed that “Loneliness for me is not as terrible as what I know other people to suffer, but because like, meditation, you know, is an inward activity. So, it helps you be with yourself and religious activity, and prayer and participating in other groups like in prayer groups, which you have, it helps. It is for me; it is both of those activities are quite important.” (#5). Social support emerged as a minor category, which was related to the caregivers gathering and communicating with friends, family, and co-workers. One specifically mentioned social support stating that “Communicating with my siblings about what was going on. I think is well, keep maintaining that balance in my life.” (#7).

### Theme 3—Loneliness

Among participants, loneliness was a frequently discussed theme related to caregiving experience. Specifically related to the caregiving, a participant stated that “I don’t know. I think the biggest take back that I find as far as the loneliness and the spiritual and caregiving is that I think people underestimate and don’t realize how lonely we really are. And it just, I think just the awareness of it, to put that awareness out there for people. And again, it’s not to say that I want recognition. It has nothing at all to say that at all. It’s just that, the humanity, emotions that I feel we need to strive for. And people don’t seem to care to do that.” (#4).

Similarly, one participant expressed their loneliness as “I feel like I’m not good enough, very low self-esteem. And now like nobody’s here for me. That’s what I feel. Like you’re not loved, like you are, you don’t have it to hold on to. I feel ugly sometimes when I feel that way [loneliness], because those are the talks that come into my mind like, ‘Oh, you are on underserving.’” (#8) Another participant referenced the loneliness in uncertainty “And during the treatments, I was always with her. So, we were kind of sharing those feelings of uncertainty and worry. We shared and helped each other get through it. So, we fortunately were able to do that. So, we got through it.” (#5).

### Theme 4—Religion to Gain Strength or Support

A prominent theme among all participants was religion to gain strength or support. All caregivers discussed religious activities to gain support and strength during their survivor’s diagnosis/treatment. Five minor categories emerged related to this theme. Specifically, when asked about religious activities such as prayer, one caregiver stated “[Prayer is] Very important. I do it at least in the morning and at night. And there’s a couple of times during the day…I try to have a conversation with God, he’s my buddy, my friend.” (#2) Specifically on the issue of faith, one caregiver stated that “Well, because you hope that the faith that you have will continue to be there for you and just one day at a time.” (#9).

Hope was an important and essential caregiving experience discussed. Hope often was described as an acknowledgment that the potential for multiple outcomes existed, and that among those outcomes are positive futures. Hope was discussed by several participants, one participant relating their experience as “That’s a hope but we are real people and I understand that is the diagnosis and I’m just, you know, what is changed is just the hope that she gets better and she doesn’t, you know, she will be in a better place when she passes.” (#8) Trust in God was revealed to be another minor category where the caregivers’ religious activities to gain support and strength during their survivors’ diagnosis and treatment regularly occurred. One participant affirmed “I try to tell her. But again, I know, I know that one day God’s going to heal her for me. He [God] is. And I just can’t wait for that day, what rejoicing that will be.” (#3) Another participant detailed his increase in faith and closeness to God became stronger from his partner’s diagnosis “But I guess, you know, I didn’t use to have conversations with God all the time. I didn’t talk to him, but I do now cause for some reason, let me tell you, I don’t know if this is, if this is right or not, but I talked to my Pastor about this and he said, you know, God knows everything. He wouldn’t get anything wrong.” (#2) One participant’s faith remained constant in her caregiving however her rate of prayers increased “Yeah. About the same [faith], but of course, you know, I do pray a lot more now for each time you have pain or when my mother calls me that he’s having an issue, of course.” (#1) Hope for the participants simultaneously occurred with connection and connection was an integral component of hope. Hope and connection to God has a reciprocity for many of the participants, the positive action of hope increased with the building and fostering of a relationship with God.

### Theme 5—Spirituality to Gain Strength or Support

The fifth emerging theme that was discussed by participants was spirituality to gain strength or support. Caregivers who discussed spiritual activities to gain support and strength during their survivor’s diagnosis/treatment discussed meditation as a way to adapt to their caregiving. Specifically, on spirituality as a social support, one caregiver stated that “But in general, being spiritual and appreciating things and feeling like that’s definitely a component and the things we’ve gone through, and helping us out, I think that’s, it’s been stronger. Like it’s gotten stronger, but not necessarily the participation in that church, you know?” (#10) One caregiver indicated that his spirituality increased during caregiving by explaining “To classify us in general, we’re not the most religious, we’re not the most religious. And we haven’t been like prior, but I’d say really, if we were more like agnostic, especially me like my position, but I’d say, and that’s like, just since forever. So, but now really, I mean, I’d say if any time we’ve been stronger or more kind of spiritual, it’s has been stronger now than it probably ever has been.” (#10).

Aspects of spirituality were referenced as a foundation for caregivers, centered around finding meaning, acceptance or reconciliation. One caregiver discussed spirituality as a foundation of life by stating “So, I’m not a religious person at all. I don’t, however we are, we’re a very strong spiritual person. So, there’s kind of a difference in that. I have a hard time conforming to manmade religion versus to just the spirituality.” (#4) Another participant’s spirituality was a source of strength though the practice of meditation “So, I’ve done yoga in the past, but it’s been for short periods of time. I have been more, I have been putting more time into the meditation practice, then I have to go do yoga.” (#7).

## Discussion

The purpose of this study was to describe Hispanic cancer caregivers’ perceptions of caregiving, spirituality, loneliness and how those relate to their HRQoL. With relatively few studies examining HRQoL in Hispanic caregivers of cancer survivors, these findings provide some insight into perceptions related to caregiving, loneliness, and spiritual experiences as they are related to HRQoL. The results of this study are consistent with those of Mytko and Knight (1999) by showing that religiosity and spirituality are associated with positive psychosocial adjustment to cancer and cancer caregiving. All participants described religiosity and spirituality contributing to their adjustment to their cancer caregiving role. Among cancer survivors and their caregivers, religious beliefs and spiritual practices may be a valuable approach to coping with the impact of cancer and related caregiving (Weaver & Flannelly, 2004). The caregivers in this study who were able to find a positive religious value in a negative situation or who found purpose in caregiving were better able to cope.

Two themes of religion and spirituality to gain strength or support that emerged from the interviews provided insight into the Hispanic cancer caregiving coping strategies. Those caregivers who experienced increased stress appeared to be motivated in the engagement of religious coping strategies. Similar to previous studies (Cupertino et al., 1998), the caregivers in this study who were better able to cope and experienced less stress, prayed more frequently and believed that religion was an important part of their life. The major category of religious coping strategies supports other findings related to loneliness and caregiving yet to be described among Hispanic caregivers (Gallegos & Segrin, 2018; Ransford et al., 2010). As in other studies (Soylu et al., 2016) the caregivers in this investigation experienced significant loneliness. To mitigate or prevent adverse health effects in Hispanic caregivers, access to high-quality, evidence-based interventions are needed, specifically developed, and tailored to supporting specific subpopulations.

Only a few studies evaluated spirituality and religiosity among Hispanic caregivers of cancer survivors (Lalani et al., 2018), to our knowledge, and none explored perceptions related to the caregiver’s loneliness among a sample of cancer caregivers on the Southwest US border that is densely populated by Mexican immigrants. The results from this study support previous research, showing that successful coping strategies for those caring for people with cancer and Alzheimer’s disease are predicted by support received from personal religious faith and number of social contacts (Rabins et al., 1990). For the participants in this study who discussed religious approaches as personal coping strategies, it appeared as a form of social support that they did not have from immediate family or friends. Spirituality and one’s religious coping strategies may be a form of social support and may fill a void when other forms of support are unavailable. Consistent with the aforementioned findings, all participants in the present study discussed religious coping strategies alongside spiritual, both of which appeared to be mentioned alongside references to improved HRQoL. Of the caregivers that discussed their organizational religiosity, such as going to church, praying, Bible study, and even virtual church attendance, religiosity was one of the more important factors responsible for successful coping with the caregiving experience. Interestingly, studies have shown that those who feel more positively about their role as a caregiver were more religious (Picot et al., 1997) in addition to having a better-quality relationship with those whom they care for (Chang et al., 1998).

The caregivers in the present study may be benefiting, in part, from their faith community wherein similar beliefs are reinforced. Furthermore, the caregivers who described their relationship with God, revealed that this relationship helped them to feel less alone and gave them hope and faith to cope with their caregiving. Those participants who disclosed more frequent organizational and intrinsic religiosity were less likely to perceive their caregiving role as burdensome. Instead, they described a sense of purpose and positive perception of life. Those with lower levels of organizational religiosity more frequently discussed negative feelings associated with their caregiving experience. The increased need for social support while confronting issues of mortality and loss in the cancer survivorship trajectory places spirituality and religiosity as a primary mechanism to help reframe the crisis and improve a sense of control. This newly improved outlook improves the caregivers experience making it less stressful, thus allowing the caregiver an improved sense of coping when encountering stress and burden while caregiving.

Our results support previous studies suggesting that Hispanic women are more inclined to be the caregivers traditionally, reflected in 60% of our small sample of caregivers (Jolicoeur & Madden, 2002; Ochoa et al., 2020). These findings suggest that the commitment to care for a person with a chronic disease such as cancer was overwhelmingly driven by our study participants who were mostly female, and the values related to the commitment to family. One caregiver expressed it as “I felt good and happy that I could help her and do things like that.” Similar family commitment was reported in a study among Asian-American women caregivers. In this study women had a devout sense of filial responsibility, similar among our caregivers (Guo et al., 2019). A sense of purpose, respect and love by giving back to a parent care recipient appeared in several interviews which supports the importance of cultural, religious and spiritual values in how these caregivers came to understand and cope with their caregiving. For many participants in this study, this practice may be guided by the cultural value of *marianismo* which is the prescription for women to be virtuous and self-sacrificing in the service and care of one’s family and loved ones (Badger et al., 2019).

Findings suggested that caregivers are less likely to engage in self-care behaviors, similar to findings from related studies in the literature (Chumbler et al., 2003; Ochoa et al., 2020; Yang et al., 2021). This was repeatedly encountered during the interviewing process; a caregiver expressed it as “there’s just some things that I had to put on hold whenever my daughter had just had a bad day so that I could be there with her.” Caregivers in this study participated in activities that would not leave their care recipient for any long length of time; coping techniques reflected this, including walking or hiking near the home, prayers, and meditation as coping strategies. These findings are comparable to those of another study of minority caregivers (Adams et al., 2002; Siefert et al., 2008). More importantly these experiences share a portrait in the Hispanic cancer caregiving trajectory and highlight the importance of understanding how Hispanics cope while providing care. Spiritual intervention can make a positive difference in a caregiver’s life and their adjustment to the cancer experience. There is a considerable need for development of programs and services that are available, affordable, and culturally sensitive to Hispanic cancer caregivers’ needs.

## Limitations

Every attempt was made to reduce weaknesses in this study; however, there are several limitations that should be considered when interpreting the findings from this study. Even though this sample of ten Hispanic cancer caregivers provides a glimpse into this new area of research, the data were collected at one point in time and may therefore not reflect the experience at different time points in the cancer caregiving continuum. This study solely interviewed English-speaking Hispanic cancer caregivers on the Southwestern border, and it is not clear if Hispanics whose spoken language is Spanish may have provided differing responses emphasizing different themes. The same may be true for Hispanics residing in other areas in the US where the context of care may generate different experiences. Finally, more women than men were enrolled. Although this reflects more general trends in caregiving, the experiences of male caregivers remain largely understudied.

## Conclusion

Interest in the psycho-socio-spiritual intersection in cancer care has increased recently, owing to the potential influence on overall health and cancer outcomes. This is particularly relevant in groups that have traditionally been highly engaged in religious and spiritual practices, such as Hispanics (Brintz et al., 2017). This study suggests that spiritual beliefs held by Hispanic family caregivers may improve their coping ability. Spirituality appears to be a central factor supporting health among this Hispanic cancer survivor-caregiver sample residing in Arizona. With Hispanics often underutilizing formal services (Crist et al., 2009), having an improved understanding of caregiving experiences will help identify strategies that are most culturally relevant, holding promise to improve HRQoL for caregivers, and thus tailor supportive care delivered to them. The development of interventions that integrate and align with cultural and spiritual beliefs may promote better health-related quality of life for cancer caregivers and the survivors they serve. This would be particularly true should interventions apply spiritual practices in the context of culturally relevant social interactions that reduce feelings of loneliness.

## Appendix: Semi-structured Interview Guide

Semi-structured interviews on loneliness and spirituality associated with being an informal Hispanic cancer caregiver

## Interview Objectives and Protocol (Tentative Areas that may be Explored)

Objectives of the interview: To answer the three research questions:

1. What are Hispanic cancer caregivers’ perceptions of loneliness?
2. What are Hispanic cancer caregivers’ perceptions of spirituality?
3. What are Hispanic cancer caregivers’ perceptions of how their loneliness and spirituality related to their HRQoL?

### Section A: Background and Establishing Rapport

Questions

1. Let’s start by you telling me a little bit about your experience of caregiving for your [partner, friend, husband/wife]?

*Prompt *How long have you been caring for your [partner, friend, husband/wife]?

2. In what ways has taking on the role of caregiving impacted your life?

*Prompt *What aspects of your life that you have had to limit as a result of your caring role?

3. Please describe what a typical day is like for you in terms of your caring role?
4. How do you feel about the way your caring role is now a significant part of your life?

*Prompt *What are your thoughts? Feelings?

### Section B: to Answer Research Questions 1, 2, and 3

Questions

1. What do you find particularly challenging about your caregiving role?
2. Tell me about when you may have experienced periods of loneliness or felt alone as a consequence of these changes to your lifestyle?

*Prompts *Could you tell me about the times when you do feel lonely?

*Prompt *What are your thoughts and feelings during these moments?

*Prompt *How long are these periods of loneliness?

*Prompt *Do you feel that the people around you are aware of your loneliness?

*Prompt *Are there any people you can talk to/confide in other than your loved one your caring for?

3. How do you manage to cope during these periods of loneliness?

*Prompt *Please describe what those are.

*Prompt *How are these helpful?

*Prompt *Are there any things that make loneliness worse?

4. How important to you is your participation in religious or spiritual activities such as going to church?

*Prompt *How often do you attend religious activities?

5. How important to you are other religious or spiritual activities such as praying or meditation?

*Prompt *How often do you practice these spiritual activities?

*Prompt *Tell me about any other religious or spiritual activities that are important to you.

6. How much has your religious or spiritual life changed as a result of your partner’s cancer diagnosis?

### Section C: Closing the Interview

That concludes our time together.

Questions

I have really enjoyed hearing about your experiences today, please tell me about any other aspects of spirituality and loneliness in caregiving that we did not talk about?
